# Discovering Digital Tumor Signatures—Using Latent Code Representations to Manipulate and Classify Liver Lesions

**DOI:** 10.3390/cancers13133108

**Published:** 2021-06-22

**Authors:** Jens Kleesiek, Benedikt Kersjes, Kai Ueltzhöffer, Jacob M. Murray, Carsten Rother, Ullrich Köthe, Heinz-Peter Schlemmer

**Affiliations:** 1 Institute for AI in Medicine (IKIM), University Medicine Essen, 45131 Essen, Germany; j.murray@dkfz-heidelberg.de; 2 Division of Radiology, German Cancer Research Center (DKFZ), 69120 Heidelberg, Germany; mail@benedikt-kersjes.de (B.K.); h.schlemmer@dkfz-heidelberg.de (H.-P.S.); 3 German Cancer Consortium (DKTK), Partner Site Heidelberg, 69120 Heidelberg, Germany; 4 Cancer Research Center Cologne Essen (CCCE), West German Cancer Center Essen (WTZ), 45122 Essen, Germany; 5 Department of General Psychiatry, Center of Psychosocial Medicine, Heidelberg University, 69115 Heidelberg, Germany; Kai.Ueltzhoeffer@med.uni-heidelberg.de; 6 Medical Faculty Heidelberg, Heidelberg University, 69120 Heidelberg, Germany; 7 Visual Learning Lab, Heidelberg University, 69120 Heidelberg, Germany; carsten.rother@iwr.uni-heidelberg.de (C.R.); ullrich.koethe@iwr.uni-heidelberg.de (U.K.)

**Keywords:** unsupervised learning, latent code, synthetic image generation, machine learning

## Abstract

**Simple Summary:**

We use a generative deep learning paradigm for the identification of digital signatures in radiological imaging data. The model is trained on a small inhouse data set and evaluated on publicly available data. Apart from using the learned signatures for the characterization of lesions, in analogy to radiomics features, we also demonstrate that by manipulating them we can create realistic synthetic CT image patches. This generation of synthetic data can be carried out at user-defined spatial locations. Moreover, the discrimination of liver lesions from normal liver tissue can be achieved with high accuracy, sensitivity, and specificity.

**Abstract:**

Modern generative deep learning (DL) architectures allow for unsupervised learning of latent representations that can be exploited in several downstream tasks. Within the field of oncological medical imaging, we term these latent representations “digital tumor signatures” and hypothesize that they can be used, in analogy to radiomics features, to differentiate between lesions and normal liver tissue. Moreover, we conjecture that they can be used for the generation of synthetic data, specifically for the artificial insertion and removal of liver tumor lesions at user-defined spatial locations in CT images. Our approach utilizes an implicit autoencoder, an unsupervised model architecture that combines an autoencoder and two generative adversarial network (GAN)-like components. The model was trained on liver patches from 25 or 57 inhouse abdominal CT scans, depending on the experiment, demonstrating that only minimal data is required for synthetic image generation. The model was evaluated on a publicly available data set of 131 scans. We show that a PCA embedding of the latent representation captures the structure of the data, providing the foundation for the targeted insertion and removal of tumor lesions. To assess the quality of the synthetic images, we conducted two experiments with five radiologists. For experiment 1, only one rater and the ensemble-rater were marginally above the chance level in distinguishing real from synthetic data. For the second experiment, no rater was above the chance level. To illustrate that the “digital signatures” can also be used to differentiate lesion from normal tissue, we employed several machine learning methods. The best performing method, a LinearSVM, obtained 95% (97%) accuracy, 94% (95%) sensitivity, and 97% (99%) specificity, depending on if all data or only normal appearing patches were used for training of the implicit autoencoder. Overall, we demonstrate that the proposed unsupervised learning paradigm can be utilized for the removal and insertion of liver lesions at user defined spatial locations and that the digital signatures can be used to discriminate between lesions and normal liver tissue in abdominal CT scans.

## 1. Introduction

Recent advances in generative adversarial networks (GANs) and other generative technologies allow for training without ground truth labels. Amongst other applications GANs [1,2] have been used for medical image synthesis, e.g., for the translation of MR into CT [3,4], PET into CT [5], or PET into MR images [6]. By employing GANs, it also has been demonstrated that lung nodules can be introduced into and removed from CT scans, leading to realistic looking images [7,8]. Related work also originates from the field referred to as anomaly detection. Here, (variational) autoencoders (VAEs) [9,10] have been used for the unsupervised lesion detection in MR scans of the brain [11,12,13].

In this paper we utilize a generative learning paradigm for the identification of digital signatures in radiological data. Apart from using them in analogy to radiomics features for the characterization of lesions, we also investigate if realistic synthetic data can be generated by manipulating these digital signatures.

Both methods, VAEs and GANs, learn a generative latent variable model of the training data and come with their respective advantages and disadvantages. Vanilla VAEs, on the one hand, often use independent Gaussian likelihood functions over individual dimensions (i.e., voxels or pixels) of the data space. Therefore, they cannot learn structured, spatially correlated noise. This often leads to a convergence of the generated predictions towards the mean of the training data, thus resulting in the generation of blurry, not very realistic samples. Furthermore, VAEs apply a variational approximation, restricting the functional family of the approximate posterior on latent variables, for a set of given observations, to Gaussian variables with a diagonal covariance matrix. Thus, by construction, they cannot learn multimodal or skewed posterior distributions, although this is remedied partially using reparameterization approaches, such as normalizing flows [14]. GANs, on the other hand, can learn any distribution on data space, which can be generated by passing some random latent variables through a deep neural network, as their training objective only relies on samples from the optimized distributions. Thus, they excel at learning structured noise and can generate very convincing samples. However, GANs do not provide a computationally efficient, direct way to map from data samples to a posterior distribution on latent variables. Recent approaches try to combine the advantages of both approaches, i.e., the flexibility of purely sampling-based optimization of GANs, and the fast amortized inference made possible by the recognition network and variational objective function of VAEs, leading to a new class of likelihood-free variational inference algorithms [15,16,17,18,19,20]. These algorithms use a similar architecture to VAEs—in terms of learning amortized generation and inference networks—when mapping from latent states to data samples and vice versa. However, they apply a learning objective, which approximates the variational free energy, using discriminator networks on data space and latent space. Thereby, they only rely on samples from all relevant distributions. This allows these approaches to combine the fast inference afforded by a variational architecture and objective functions with the flexibility of an approach that does not have to make strong assumptions about the functional family of the likelihood or posterior. This leads to powerful and very flexible architectures that are able to generate realistic medical images and have an accessible latent code that can be exploited for downstream tasks. In our case, this latent code vector captures a compressed version of the structures that are present in the liver image patches. It can be used not only for the generation of (novel) output images but also as a feature vector for radiomics analyses, making the classification of different tissue types possible. Hence, we speak of “digital tumor signatures” when referring to this latent code vector.

In the literature, at least four generations of radiomics are described [21]. The first two generations incorporate handcrafted features in conjunction with classical machine learning methods. The second generation is characterized by the extraction of a multitude of generic handcrafted features and currently still represent the primary radiomics approach. In the 3rd generation deep learning (DL) models are utilized to extract features, thereby exploiting that DL architectures can learn representations without relying on feature engineering. This should lead to better predictions as it allows to learn complex, non-linear relationships within the data. End-to-end approaches are considered 4th generation radiomics. These approaches combine learning of representations and classification, e.g., distinguishing clinical endpoints or genetic traits, in a single architecture. An example is the study of Hosny et al. that demonstrate this approach for lung cancer prognostication [22]. A disadvantage of an end-to-end solution that is often stated is that the processes leading to the output are more difficult to comprehend and interpret.

In a recent approach, coined “deep radiomics”, Kobayashi and colleagues proposed to constrain the internal variability of convolutional neural networks through vector quantization [23]. They demonstrate that the learned internal feature representations can be exploited as imaging markers that in turn can be used for glioma grading.

This approach is similar to our digital signatures approach. Both methods could be categorized as 3rd generation radiomics. However, our solution has several advantages. Training is conducted in an unsupervised fashion, we utilize a generative instead of a discriminative approach, allowing for generating synthetic data and as mentioned above, do not need to constrain the expressiveness of the learned distribution as it is done with vector quantization.

In this article, we present a generative architecture for learning of spatially encoded digital tumor signatures. To the best of our knowledge, no comparable approaches have been described in the literature. To evaluate the model, we use abdominal CT scans containing liver lesions. We investigate how direct alteration of the latent code allows for the generation of synthetically manipulated high-quality radiological data, i.e., how to remove or insert liver lesions at a given spatial location and if medical experts are able to distinguish the synthetic images from real data. Further, we analyze if the digital signatures can be used to discriminate between lesions and normal appearing liver tissue.

## 2. Materials and Methods

### 2.1. Data and Preprocessing

The inhouse training set consisted of abdominal CT scans from 57 patients in the portal-venous phase; 32 contained at least one lesion, the remaining 25 scans displayed no visible liver lesion. With lesions we allude to either benign or malignant changes of the liver tissue.

The spiral images were acquired during routine clinical workup on a 2 × 64-slice dual source dual energy CT (Siemens Somatom Definition Flash, Siemens AG, Forchheim, Germany), using two different tube voltages (100 kV and tin filtered 140 kV, reference tube currents 200/155 mAs). The two images were fused to a virtual image of 120 kV with a weighting factor of 0.5. Using a standard soft tissue kernel (B31f) 3 mm axial slices were reconstructed with an in-plane pixel spacing ranging from 0.65 to 0.97 mm. The portal venous images were acquired 60 s after intravenous application of nonionic iodinated contrast agent (Imeron 300, Bracco, Konstanz, Germany) with a body weight adapted amount and flow rate.

In addition, we used the publicly available LiTS data set containing 131 scans that originated from 5 different institutions and was annotated by experts as described in Bilic et al. [24]. The heterogenous LiTS imaging data were acquired with different CT scanners and acquisition protocols, leading to distinct differences in resolution and image quality. The axial resolution varies from 0.56 to 1.0 mm and the z- spacing from 0.45 to 6.0 mm. No information is given on the contrast agent administration, but visual inspection reveals the presence of some arterial phase images.

The CT volumes were used without resampling or normalization and split into patches of 96 pixels × 96 pixels by sliding a two-dimensional window over each axial slice. A step size of 48 pixels was used and extracted patches had to contain liver tissue, resulting in approximately 500,000 patches in total. The HU values of the patches were clipped to a range between −110 and 190 HU and scaled to be in the range [−1, 1]. From the LiTS data set we created balanced test sets with the same number of normal and lesion patches, by randomly selecting 1024 patches for the principal component analysis (PCA) embedding and 1000 patches for the classification experiment.

### 2.2. Model Architecture and Training

**Figure 1:**
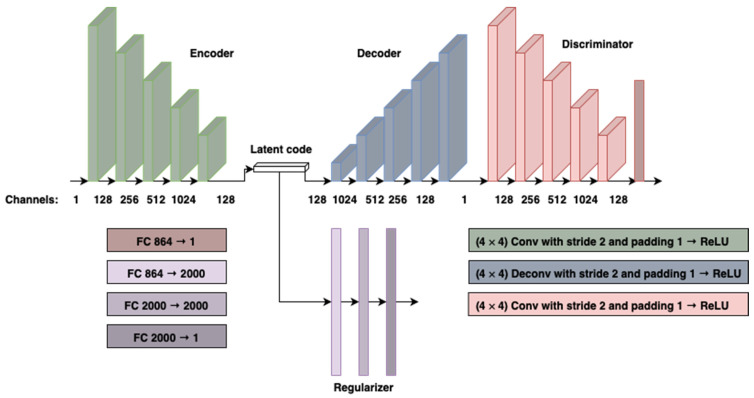
Model architecture of the IAE. Encoder and decoder are fully convolutional. The discriminator has both convolutional and fully connected layers. The regularizer is built of three fully connected layers. Abbreviations: FC—fully connected layer, Conv—convolutional layer, ReLU—rectified linear unit.

The architecture (Figure 1) is an extension of the IAE architecture [17] with a fully convolutional encoder and decoder.

We augmented the previously proposed loss function of the decoder by an additional pixel-wise L1 term, i.e., the absolute difference between the predicted and true value, which was weighted by a factor of 500 to account for approximately 80% of the total loss value. Empirical analysis yielded that this was necessary to obtain reconstructions as similar as possible to the original images. In the original architecture the latent code was divided into a global and local part. It was shown that the global part captures general information whereas the local part introduced stochasticity for variations of the output. In our experiments we removed the local code, thereby turning this part of the architecture into a deterministic autoencoder. The reasoning is that the reconstructions should be as realistic as possible and we wanted to deterministically modify regions of the image by directed modifications to the latent code. For this purpose, we additionally extended the latent code by a third dimension. The first two dimensions correspond to the spatial locations of the input patch, while the third dimension, i.e., the channel dimension, encodes the information for a specific location. We chose the latent code to be of size 3 × 3 × 128. The latent space was adversarially constrained to be close to a standard normal distribution with zero mean and unit variance. This normalization in latent space allowed the architecture to learn a full generative model of the data distribution, which could be used to generate samples representative of the training distribution, or to automatically detect outliers by quantifying the prior probability of embedded data in latent space. To further improve the quality of reconstructions, we turned the AE into a denoising AE by using an input noise vector sampled from a Gaussian distribution with zero mean and variance of 0.1 [25]. The input noise vector was not used during the prediction.

The model was trained for 400 epochs using the Adam optimizer with an initial learning rate of 0.0002, which was reduced to 0.00002 after 200 epochs. For data augmentation, random rotations and up–down/right–left flipping were applied with equal probability. Training took approximately 12 h on a desktop PC with an 11 Gb NVIDIA Geforce RTX 2080 Ti GPU.

### 2.3. Evaluation of Synthetic Images

To evaluate the visual appearance of the synthetically generated images, we conducted a survey with five radiologists. Their radiological experience ranged from less than one year to over 30 years with a median experience of 3 years.

Using a custom browser-based presentation tool two image patches were presented next to each other to the participants. We measured the classification accuracy and the decision time for each participant. They only received minimal instructions and were not informed about the time measurements. For each experiment we used 40 patches from the inhouse data. In the first experiment 20 synthetic images had a lesion that was inserted into a previously patch of normal liver tissue and the remaining 20 synthetic images were modified such that a real lesion was removed. Modified patches were selected manually. That means, only such patches were selected that contained a lesion in a suitable position for being removed or that provided a suitable spot for inserting a lesion. However, once the selection was fixed, the modification was applied without discarding patches that showed any artifacts or abnormalities, aiming for a patch selection as unbiased as possible for the first experiment. The data for the second experiment consisted of 40 randomly selected patches.

To assess the inter-rater reliability, we computed Fleiss’ kappa. We modeled the experiments as binomial processes to determine if rater decisions were above the chance level for a given significance level defined with *p* < 0.05. Further, by combining the answers using majority voting we obtained an ensemble rater that was also tested against chance level. To assess the difference in reaction times we used two-sided *t*-tests, deliberately without correction for multiple comparison (Appendix A).

### 2.4. Latent Code Lesion Classification

To investigate the differences of encodings from normal and lesion patches we followed two strategies. For visual inspection of the learned structure, we reduced the latent code to two dimensions using principal component analysis. Additionally, we trained different classifiers to distinguish between healthy and diseased patches in the latent space, assuming that classification accuracies are indicative for separability in the latent space. For this purpose we used the sklearn version 0.20.3 implementations of LinearSVM, random forest (RF), multi-layer perceptron (MLP), and naive Bayes [26]. For the RF we used 1000 trees with a maximum depth of 10. For all other methods we kept the default parameters (listed in Appendix B). We ran a 10-fold cross validation and trained all classifiers on the flattened latent space.

## 3. Results

### 3.1. Latent Code Manipulation

We distinguish three different types of images: original, reconstructed, and synthetic. The term original denotes the acquired CT image prior to feeding it into the model, reconstructed refers to the deterministic output of the model, i.e., the encoded and decoded image without any modifications of the latent code, and synthetic describes the artificially altered image by adjusting the latent code.

**Figure 2:**
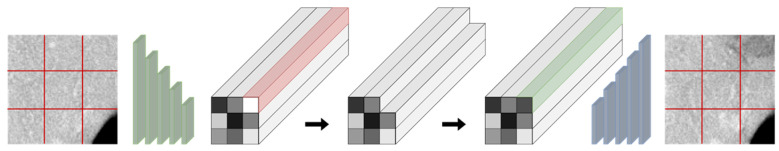
Schematic for latent code manipulation. Each shown CT patch is 96 × 96 pixels in size, corresponding to an approximate physical size of 96 × 96 mm.

For adjustments to the latent code, we replaced the channel information of the latent code for a given spatial location with the channel information of a spatial location from another patch (Figure 2).

**Figure 3:**
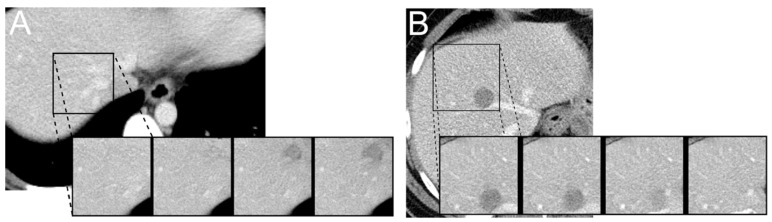
Synthetic insertion (**A**) and removal (**B**) of a liver lesion. The four insets each display generator output and a linear interpolation of latent code with 25, 75, and 100% from left to right. Each inset is 96 × 96 pixels in size, corresponding to an approximate physical size of 96 × 96 mm.

Specifically, to generate synthetic images with and without lesions, the latent code of a lesion patch was replaced with the latent code of normal liver tissue and vice versa. This enabled us to “convert” regions containing lesions to normal liver tissue and to insert lesions into selected, previously normal appearing liver regions. This can be done for any spatial location on a given patch. By design, the first two dimensions of the latent code determine the spatial location whereas the third dimension encodes what we refer to as a digital tissue signature, i.e., the encoding of the latent code as it was learned by the model. This digital signature can be manipulated resulting in images that are hard to distinguish from real data, since the decoder learned to combine several spatial locations to a single perceptually consistent image by adversarial training. Examples of synthetically modified images are shown in Figure 3.

Further, by linearly interpolating between a digital signature encoding normal tissue and a signature encoding a lesion (and vice versa) we were able to gradually insert and remove lesions at given spatial locations (see Videos S1 and S2, Supplementary Materials). For instance, this could be used to synthetically generate different stages of lesion growth resulting in CT scans with different levels of visual discernibility.

### 3.2. Evaluation of Synthetic Images

To determine the quality of our results, we conducted two surveys with five radiologists. For the first experiment, they were asked to distinguish between the synthetic and original image. In the second experiment, they had to distinguish between reconstructed images and original images. The results (Table 1) indicate that the experts were neither able to differentiate with sufficient confidence between the synthetic and original nor between the reconstructed and original image patches.

For both experiments they achieved accuracy scores with an average accuracy of 0.635 and 0.57 respectively. From the modeled binomial process we were able to infer the chance level interval for the accuracy to be in the range of [0.35, 0.65]. For experiment 1, only rater 5 and the ensemble rater were marginally above the chance level. For the second experiment, no rater was above chance level. The results were independent of the experience level of the raters. There were significant differences between some experts w.r.t. the time they needed for a decision (see Supplementary Materials). However, the reaction times did not impact accuracy. It should be noted that the discriminator could not be fooled, indicating that the adversarial training was successful.

### 3.3. Latent Code Lesion Classification

**Figure 4:**
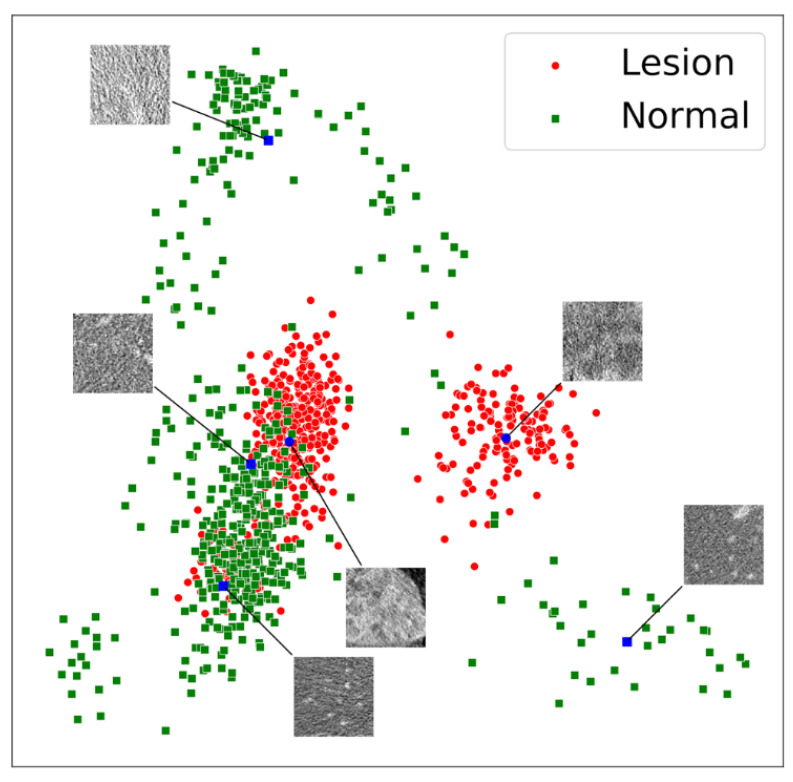
Latent space visualization after PCA utilizing the first two principal components. The model was trained on the inhouse data containing patches with normal appearing liver tissue (green) and lesions (red). Evaluation was performed on the external LiTS data. The insets depict 6 example patches out of 1024 randomly chosen raw image patches. Each patch is 96 × 96 pixels in size, corresponding to an approximate physical size of 96 × 96 mm.

Aside from using the learned digital signatures for synthetic image generation we also exploited them to determine whether a given patch contained a lesion. For this purpose, we used PCA to reduce the high dimensional latent space into two dimensions. No normalization of the latent space was necessary as it is constrained to be close to a standard normal distribution with zero mean and unit variance during training. The inherent structure was visible and distinct clusters could be easily discriminated (Figure 4).

The discriminability increased even further, when training was conducted only with patches that did not contain any lesion.

To confirm and quantify the visual results we trained four different out-of-the box classifiers on the latent space obtained with inhouse data for discriminating normal tissue from tissue patches that contain lesions. This led to a classification accuracy on the LiTS data of up to 95% (sensitivity of 94% and specificity of 97%) with a LinearSVM when utilizing patches with normal appearing liver tissue and lesions for constructing the latent space (Table 2, all data). The MLP achieved comparable scores (95% accuracy, 93% sensitivity, and 98% specificity). Using only normal appearing liver tissue for training of the digital signatures, the accuracy of the LinearSVM could be improved further, yielding up to 97% accuracy, a sensitivity of 95%, and a specificity of 99%.

## 4. Discussion

We propose to use a combination of a denoising autoencoder and two GAN-like units for unsupervised learning of 3D latent representations, encoding image content together with its spatial location. We refer to these encodings as digital signatures and demonstrate two possible applications on abdominal liver CT scans: (i) they can be manipulated to insert and remove liver lesions in order to generate 2D image patches that to human experts appear convincingly realistic and (ii) they can be used to discriminate between normal appearing liver tissue and tissue containing lesions.

It should be stressed that the model was trained on a homogeneous internal data set and evaluated on external data, which exhibits drastically different imaging characteristics due to heterogeneous acquisition parameters, contrast agent administration, and scanner vendors. Next to clipping and scaling the data, no additional preprocessing of the data was necessary. Moreover, the internal data set only contained 57 or 25 CT scans, depending on the experiment. Yet, training and results proved to be robust as demonstrated by the evaluation on the public data set.

The presented line of research combines several subfields currently explored in medical imaging: image synthesis, anomaly detection, and disentanglement. Whereas current literature usually focuses on a single topic, we combine the discoveries. This is achieved by focusing on the digital signatures, i.e., the latent code, as common ground.

Mirsky et al. proposed to use conditional GANs for insertion and removal of lung nodules to tamper with 3D CT scans in a malicious attack scenario within clinical IT infrastructures [8]. They use two distinct GANs, one for the insertion and the other one for the removal of nodules. Apart from a single architecture being arguably more elegant, this also requires prelabeling of images for training as being benign or malignant. This is not necessary in our approach. For the location specification to be altered, they determine a candidate voxel and cut out a cuboid region of size 32 × 32 × 32 around it. Post-processing is necessary, like histogram equalization and Gaussian blurring, so it can be seamlessly blended into the surrounding anatomy. In contrast, by altering the latent space, our approach leads to a perceptually consistent image by design.

Using a multiconditional GAN for synthetic lung nodule generation in CT scans, Han et al. were able to obtain realistically looking image patches of size 32 × 32 × 32 [7]. They introduced two discriminators, one to distinguish between real and synthetic nodules within noise box-centered surroundings, the other based on size/attenuation conditions. In our approach the attenuation can be tuned by interpolating between different digital signatures. They also report that GAN training with an improved data augmentation ratio instead of L1 loss leads to better performance. This is contrary to our observation where L1 loss was essential for robust, high quality generation of images. This discrepancy will be investigated in future projects.

Several approaches have been presented for the classification of CT liver lesions [27,28,29,30,31]. They have in common the lesion that first needs to be delineated. The classification then relies on handcrafted features, e.g., texture or histogram features. Frid-Adar and colleagues used a CNN for the classification [32]. For training purposes, they used GAN variants to generate patches of size 64 × 64.

A comparison with other results reported in the literature is not possible directly. Different data sets have been used and different class labels have been considered [27,28,29,30,31,32]. Reported accuracies range from 81.7% to 97%. The highest reported sensitivity and specificity values were 85.7% [32] and 93.6% [31], respectively. We yield 97% accuracy, 95% sensitivity, and 99% specificity with unsupervised training solely on normal appearing liver tissue and without prior segmentation or delineation of the lesion. Yet, we only considered two separate classes. We would like to stress that the main goal of our approach was not to distinguish liver lesions. Instead, it should be considered as a proxy experiment, together with the visual Turing test, to demonstrate that suitable “digital signatures” can be learned. Next to the generation of synthetic patches, these can be utilized for discrimination without substantial effort, i.e., tuning a specific machine learning method to yield optimal performance.

Based on the presented results, we conjecture that the digital signatures learned by unsupervised training, next to classification, can be exploited as radio(geno)mics features. For this purpose, disentanglement methods could help to understand the structure of the latent code and to identify components that are indicative of normal and pathologic observations. For instance, informative latent variables have been discovered that encode the style of handwritten letters and digits by structuring the latent space with nonlinear independent component analysis [33]. Chen and colleagues learned representations using an autoencoder and clustered them with a Gaussian mixture model [34]. However, they used canonical radiomics features as an input to the model. The proposed pipeline was evaluated on 108 MRI scans of patients with liver metastases originating from colorectal cancer, assigning patients to clusters of differing survival times. Recently, Song et al. proposed to use DL-derived semantic features, akin to our digital signatures, to identify patients suffering from non-small cell lung cancer that will not benefit from a specific therapy [35]. They utilized the BigBiGAN network for their experiments [36].

For our approach, additional potential applications are conceivable, e.g., it could be utilized for the education of medical students and radiologists in training. By interpolating the latent space only faintly visible lesions could be generated for educating the “eye” of the reader (Figure 3), training them for higher accuracy at the border of perceptibility. Since the position of the lesion can be specified automatically with our method, performance could be evaluated, e.g., by clicking on the lesion as soon as it becomes visible—without any manual intervention.

An ideal method for synthetic data generation should not require large amounts of data for training. However, this is usually required for GANs to capture the data distribution well. In our experiments we deliberately decided to avoid the generation of synthetic data based on random noise vectors. Instead, we took the learned digital signatures of real lesions and recombined them to generate new lesions at arbitrary locations. This led to authentic lesions, while using only a few training examples.

Future research will address current limitations of the proposed framework. So far, we evaluated the method only on 2D data patches. Due to anatomical consistency the task is more challenging in 3D and also requires more GPU-RAM. Additionally, we only demonstrated the applicability of the method for a single tissue type and modality. The limitations will be addressed in future experiments. These will be conducted using 3D patches originating from different anatomical regions and acquired with different imaging modalities, e.g., also including MR images. To enforce anatomical consistency adjustments to the architecture might be necessary. Further, we will investigate disentanglement methods to enhance the explainability of the latent space, which is necessary for potential clinical applicability.

## 5. Conclusions

In conclusion, we present the robust application of an unsupervised learning paradigm for the removal and insertion of liver lesions at user defined spatial locations. Further, we demonstrate that the learned digital signatures can be used to discriminate lesions from normal liver tissue with high accuracy.

## Figures and Tables

**Table 1:** Results of the visual Turing test (std indicates ± 1 standard deviation, *n* = 40).

Rater	Accuracy Experiment	Times s (std) Experiment 1	Accuracy Experiment 2	Times s (std) Experiment 2
1	0.65	8.05 (4.71)	0.425	5.03 (2.9)
2	0.625	14.34 (13.53)	0.575	7.84 (5.84)
3	0.575	7.62 (13.99)	0.6	4.01 (3.67)
4	0.6	9.86 (6.65)	0.6	6.21 (4.5)
5	0.725	72.3 (216.61)	0.65	15.29 (9.48)
Ensemble	0.7	-	0.65	-

**Table 2:** Classification accuracies of digital signatures. ((std) indicates ± 2 standard deviation, ACC accuracy, SE sensitivity, SP specificity, AUC area under the curve, *n* = 1000 patches.)

Classifier	All Data				Normal			
	ACC	SE	SP	AUC	ACC	SE	SP	AUC
Linear SVM	0.95 (0.02)	0.94 (0.04)	0.97 (0.01)	0.96 (0.02)	0.97 (0.02)	0.95 (0.03)	0.99 (0.02)	0.97 (0.02)
Random Forest	0.87 (0.04)	0.93 (0.04)	0.80 (0.06)	0.86 (0.04)	0.91 (0.04)	0.92 (0.06)	0.89 (0.04)	0.90 (0.04)
MLP	0.95 (0.03)	0.93 (0.05)	0.98 (0.03)	0.95 (0.02)	0.96 (0.02)	0.93 (0.04)	0.99 (0.02)	0.96 (0.02)
Naive Bayes	0.84 (0.05)	0.92 (0.05)	0.76 (0.09)	0.84 (0.05)	0.88 (0.04)	0.91 (0.06)	0.84 (0.06)	0.88 (0.04)

## Data Availability

The publicly available datasets analyzed in this study can be found here: https://competitions.codalab.org/competitions/17094, accessed on 2 November 2018.

## Supplementary Materials

The following are available online at https://www.mdpi.com/article/10.3390/cancers13133108/s1, Video S1: [animation_insert_lesion.mov]. Synthetic insertion of a lesion at different locations of the same patch. The “digital signature”, i.e., the third dimension of the latent code, is taken from a lesion patch and inserted at a chosen spatial location of normal appearing liver tissue. It is linearly inter-polated between the original and altered latent code for generating this animation, Video S2: [ani-mation_remove_lesion.mov]. Synthetic removal of two lesions. The “digital signature”, i.e., the third dimension of the latent code, is taken from a normal patch and inserted at the spatial location of the lesion. It is linearly interpolated between the original and altered latent code for generating this animation.

## Appendix A—Statistical Analysis

The statistical analyses were performed using custom python scripts. Below the results of the two sided *t*-tests for the comparison of reaction times are given. Asterisks indicate a significance level of *p* < 0.05.

Experiment 1

Rater 1 vs. Rater 2 (statistic = −2.7420538784160478, *p* value = 0.007569154296333987) *

Rater 1 vs. Rater 2 (statistic = 0.1820547772716781, *p* value = 0.8560120482099625)

Rater 1 vs. Rater 2 (statistic = −1.3866046545098654, *p* value = 0.1695127800979864)

Rater 1 vs. Rater 2 (statistic = −1.8521007888269003, *p* value = 0.06779495429447562)

Rater 2 vs. Rater 3 (statistic = 2.1570553441546894, *p* value = 0.0340805997172787) *

Rater 2 vs. Rater 3 (statistic = 1.8564265023707884, *p* value = 0.06716890115419788)

Rater 2 vs. Rater 3 (statistic = −1.6678510940888802, *p* value = 0.09935541002215911)

Rater 3 vs. Rater 4 (statistic = −0.9032535586447072, *p* value = 0.36917244828206985)

Rater 3 vs. Rater 4 (statistic = −1.861069472879768, *p* value = 0.06650233614344676)

Rater 4 vs. Rater 5 (statistic = −1.7995055802104227, *p* value = 0.0758059791388329)

Experiment 2

Rater 1 vs. Rater 2 (statistic = −2.685734151471288, *p* value = 0.008838749480933973) *

Rater 1 vs. Rater 2 (statistic = 1.3632679062509367, *p* value = 0.17672031901173887)

Rater 1 vs. Rater 2 (statistic = −1.3765153811881174, *p* value = 0.17260079372736484)

Rater 1 vs. Rater 2 (statistic = −6.462580475410759, *p* value = 8.137130260702532 × 10^−9^) *

Rater 2 vs. Rater 3 (statistic = 3.4631820348205937, *p* value = 0.0008698600759136433) *

Rater 2 vs. Rater 3 (statistic = 1.3772858777094696, *p* value = 0.17236346640916345)

Rater 2 vs. Rater 3 (statistic = −4.180303065472304, *p* value = 7.544941128344643 × 10^−5^) *

Rater 3 vs. Rater 4 (statistic = −2.3677053810044373, *p* value = 0.020376584836282633) *

Rater 3 vs. Rater 4 (statistic = −6.9294854378836135, *p* value = 1.0726186719247145 × 10^−9^) *

Rater 4 vs. Rater 5 (statistic = −5.4044324519727915, *p* value = 6.858611514979791 × 10^−7^) *

## Appendix B—Classifier Parameters

For the LinearSVM the l2-penalty and squared_hinge loss were used, tolerance for the stopping criterion was set to 0.0001 and the regularization parameter C was set to 0.025. The MLP had two hidden layers with 100 neurons each, ReLU as an activation function and Adam as optimizer were used. The random forest had a maximum depth of 10 and only 1 feature was considered for splitting. The Gaussian naive Bayes classifier with no priors was used.
